# Family Resilience and Psychological Responses to COVID-19: A Study of Concordance and Dyadic Effects in Singapore Households

**DOI:** 10.3389/fpsyg.2022.770927

**Published:** 2022-03-01

**Authors:** Yi-Ching Lynn Ho, Mary Su-Lynn Chew, Dhiya Mahirah, Julian Thumboo

**Affiliations:** ^1^Centre for Population Health Research and Implementation, Singapore Health Service, Singapore, Singapore; ^2^Programme in Health Services and Systems Research, Duke-NUS Medical School, Singapore, Singapore; ^3^Department of Rheumatology and Immunology, Singapore General Hospital, Singapore, Singapore; ^4^Medicine Academic Clinical Program, Duke-NUS Medical School, Singapore, Singapore

**Keywords:** COVID-19, family resilience, threat perception, psychological impact, risk factors, protective factors, actor-partner interdependence model, family dyads

## Abstract

The impacts of COVID-19 may be magnified in a shared environment like the household, especially with people spending extended time at home during the pandemic. Family resilience is the ability of a family to adapt to crisis and can be a protective factor against stress and negative affect. While there have been calls to address family resilience during the pandemic, there is a lack of empirical study on its benefit. In this dyadic observational study, we sought to investigate the concordance of family members’ psychological responses to COVID-19, whether dyad members’ risk factors (COVID-19 exposure and financial impact) mutually affected each other’s psychological responses, and importantly, whether family resilience was a significant factor in these responses. A total of 200 family dyads from the same household completed the Family Resilience Assessment Scale and questionnaires on COVID-19 threat perception, impacts, and exposure. We found concordant dyad responses for COVID-19 threat perception, but not for psychological impact. Using the Actor-Partner-Interdependence Model framework, we found that one’s psychological impact was affected by the financial impact from both dyad members. After controlling for risk factors and demographic covariates, we found that family resilience significantly associated with lower COVID-19 psychological impact, though not with threat perception. The findings suggest that both family and individual factors need to be addressed and there may be benefit in addressing multilevel risk and protective factors using an ecological systems approach, which may help prepare the population for future crises.

## Introduction

In a pandemic and global crisis like COVID-19, the role of the family unit (Sharma, 2013) and its collective reactions have come into prominence (Masten and Motti-Stefanidi, 2020; Prime et al., 2020; Rolland, 2020). The impacts of COVID-19 on loss of life, health (physical and mental), livelihoods and the uncertainties, and stress of disruptions to daily routines may be magnified in a shared environment like the household (Walsh, 2020), especially with people spending extended time at home due to movement controls and closure of community facilities like schools. For instance, Canadian parents with children living at home reported worse mental health as a result of the pandemic (Gadermann et al., 2021), while there has been increased depression and anxiety among Chinese individuals associated with a family member being a healthcare worker (Ying et al., 2020), and psychological distress in Japanese individuals associated with a family member or friend diagnosed with the virus, compared to positive diagnoses of known contacts in proximal settings (Tanoue et al., 2020).

These observations point to the relational network of the family, where losses or stressors experienced by members of the family may reverberate through the network (Walsh, 2020). These findings highlight the need and necessity for the family to adapt to crises. Adaptation comes from the interplay of family vulnerability, risk, and resilience as multilevel recursive influences in the systems perspective (Walsh, 2020). Family resilience can be seen as the ability of a family to adapt to crisis and disruption (Walsh, 1996), and can be a protective factor against stress and negative affect (Brivio et al., 2021). In a family resilience framework, key processes in family functioning mediate the recovery and resilience of vulnerable members as well as the family unit (Walsh, 1996, 2003). The key family processes include the domains of family belief systems, organization patterns, and communication processes. In a severely disruptive situation like COVID-19, it will be important to understand how families buffer and manage stress. It will also be important to understand how to build up resilience not just in individuals, but also in families as fundamental units in society to face future crises. While calls for building up family resilience have been made (Masten and Motti-Stefanidi, 2020; Prime et al., 2020; Walsh, 2020), there has been little empirical research on family resilience as a protective factor during the pandemic.

Current research on coping with COVID-19 has focused largely on the role of individual protective factors, with less attention given to the influence of the socio-ecological systems surrounding an individual, such as the family (Masten and Motti-Stefanidi, 2020). The importance of the family’s role in mitigating negative mental health during the pandemic has nonetheless been suggested in a few studies. For example, in a survey of Canadian workers during COVID-19, family functioning and social support appear to be protective factors for mental health and wellbeing, alongside personal factors like individual resilience, social participation, and trust in healthcare institutions (Coulombe et al., 2020). In patients dealing with cancer during COVID-19, the protective effect of family resilience was enhanced by the locus of control in mitigating negative affect associated with the pandemic (Brivio et al., 2021). In these studies, assumptions were that the individuals’ ratings on family factors were representative of the family and that there was standard exposure to COVID-19, even though both physical (Ding et al., 2021) and media-related exposure (He et al., 2021) may exacerbate psychological responses to the pandemic.

Based on family dyads recruited from the community, this study aimed to investigate the following research questions: First, how concordant are family members’ psychological responses in relation to COVID-19 (specifically, perceptions of COVID-19 threat and psychological impact due to COVID-19)? If psychological responses have coherence within the family, it would highlight the value of addressing psychological responses on the family level, to complement existing research which has mainly been focused on the individual. Second, does a family member’s COVID-19 exposure or financial difficulties affect another member’s psychological response? Using these two known personal risk factors, this question aims to investigate the mutual influences of family members, which have largely been assumed (Tanoue et al., 2020; Ying et al., 2020). Third, is family resilience a significant factor in mitigating the psychological responses to COVID-19 after accounting for COVID-19 exposure? Findings will contribute to the scant empirical research on family resilience as a protective factor during the pandemic. Combined with the first two questions, the overall research may provide impetus for building families resilient to the next crisis.

The corresponding hypotheses were as: (1) Family dyad members will demonstrate at least moderate agreement [intraclass correlations (ICC)≥0.5] on COVID-19 psychological responses; (2) There will be significant “partner effects” of COVID-19 risk factors (exposure and financial impact) on a member’s COVID-19 psychological response; and (3) COVID-19 psychological responses will be negatively associated with the level of family resilience, after accounting for the risk factors of COVID-19 exposure and financial impact.

## Materials and Methods

We performed a cross-sectional survey in Singapore over a 12 week period (25th October 2020 to 15th January 2021). Prior to the study, a partial national lockdown (“circuit breaker”) was in place from 3rd April to 1st June 2020, after which the country moved in phases toward resuming activities with a decrease in virus transmission rates: Phase 1 (2nd to 18th June 2020) allowed the resumption of low-risk and essential activities. Phase 2 (19th June to 27th December 2020) allowed more businesses and social activities to resume with safety measures, and Phase 3 (28th December 2020 to 31st March 2021) marked the resumption of most activities along with the start of vaccine administration to the population. This study took place during Phases 2 and 3 when the virus transmission rate within the community was low.

The study was approved by the institutional ethics committee (CIRB Ref. 2020/2195).

### Participants and Procedures

A total of 200 family dyads (i.e., 400 individuals) living in the same household were recruited through convenience sampling from a survey panel maintained by a commercial research company, as well as from advertisements placed on social media (Facebook). Due to COVID-19 restrictions and the prevailing research directives, these remote recruitment methods were used in order to reach out to a larger number of potential participants without the need for physical meetings or the mailing of paper forms. Members of the survey panel (who had consented to being contacted for study recruitment) were invited through email and the response rate was 6.7%. To reach out to a wider group, Facebook advertisements were placed. Individuals who registered interest through either platform were asked if they had a family member who would be interested to participate together with them and had agreed to provide their contact details. Each person was contacted separately to provide study details and determine eligibility. We included only dyads who were family members living in the same household, at least 15 years of age, and were citizens or permanent residents. Each eligible dyad member was emailed a unique weblink for the online survey and was instructed to complete the survey independent of the other member. Of the individuals who started the survey, the completion rate was 94.6%. A total of 3.4% (16 sets) of the completed surveys were discarded upon quality checks by the survey company (which included checking for nonsense responses (e.g., same answer throughout), speeding through the survey, and overseas IP addresses). We continued recruitment until we achieved 200 valid sets of dyad responses.

### Measures

The self-reported measures used in the online survey are outlined below. They were in the English language. Data on demographic characteristics of participants (age, gender, race, education, occupation, marital status, type of housing, and household income) were also collected.

#### COVID-19 Questionnaires

The study variables of COVID-19 perceived threat, psychological impact, financial impact, and exposure were measured using the short version of the questionnaires designed by Conway et al. (2020) and listed in Table 1. Ratings are made on a 7-point Likert scale (1 = “not true of me at all” to 7 = “very true of me”). Higher scores indicated higher levels of perceived threat, impacts, or exposure.

**Table 1:** Descriptive statistics of the domains.

Variable	Mean	SD	Min	Max
*COVID-19 threat perception* *^a^*	10.62	5.28	3	21
Thinking abocut the coronavirus (COVID-19) makes me feel threatened I am afraid of the coronavirus (COVID-19) I am stressed around other people because I worry I’ll catch the coronavirus (COVID19)				
*COVID-19 psychological impact* *^a^*	4.84	3.35	2	14
I have become depressed because of the Coronavirus (COVID-19) The Coronavirus (COVID-19) outbreak has impacted my psychological health negatively				
*COVID-19 financial impact* *^a^*	6.14	3.76	2	14
The Coronavirus (COVID-19) has impacted me negatively from a financial point of view I have lost job-related income due to the Coronavirus (COVID-19)				
*COVID-19 exposure* a	11.93	4.28	6	26
I have been diagnosed with coronavirus (COVID-19). I have had coronavirus-like symptoms at some point in the last two months I have been in close proximity with someone who has been diagnosed with coronavirus (COVID-19) I have been in close proximity with someone who has had coronavirus-like symptoms in the last two months I watch a lot of news about the Coronavirus (COVID-19) I spend a huge percentage of my time trying to find updates online or on TV about Coronavirus (COVID-19)				
*Family resilience assessment scale (FRAS)* *^b^*	167.76	17.67	122	210
Meaning-Making and Positive Outlook (13 items)	41.95	5.48	21	52
Transcendence and Spirituality (Four items)	10.98	3.20	4	16
Flexibility and Connectedness (10 items)	31.90	4.05	22	40
Resources-Community (Eight items)	24.15	3.54	9	32
Resources-Neighbors (Two items)	4.83	1.50	2	8
Clarity and Open Emotional Expression (10 items)	31.12	4.65	15	40
Collaborative Problem Solving (Seven items)	22.59	3.23	12	28

*SD: Standard deviation. Numbers rounded to 2 decimal places*.

a Conway et al. (2020).

b Chew and Haase (2016).

The domain of Perceived Threat was assessed with three items (score range: 3–21), measuring concerns about COVID-19, such as threat, fear, and stress due to the virus. The domain of Psychological Impact was measured with two items (score range: 2–14), looking at impact on depression and psychological health. The domain of Financial Impact contained two items (score range: 2–14), assessing impact on finances and income due to COVID-19. Confirmatory factor analysis indicated strong factor structures for the scales. (The factors of Psychological Impact and Financial Impact were part of a larger 3-factor Impacts scale.) The scales had good face validity and strong internal reliability within each factor (Conway et al., 2020). We also corroborated the good internal reliability within these factors using data from this study (COVID-19 Perceived Threat: 0.92; COVID-19 Psychological Impact: 0.93; and COVID-19 Financial Impact: 0.77).

We used six items (score range: 6–42) from the COVID-19 Experiences scale from Conway et al. (2020) to assess the level of exposure (physical and media-related) to COVID-19 (Table 1). The items on personal diagnoses/symptoms and proximity to cases assessed the level of direct (physical) exposure, while items on experiencing news on COVID-19 provided a measure of indirect (psychological) exposure through media. With this array of items, the scale is mainly descriptive and there is no specific latent construct. In line with this, internal consistency was not strong (0.57 Cronbach alpha based on this study). Nonetheless the individual items were tested to have face validity (Conway et al., 2020). The recommendation from Conway et al. (2020) is to treat the items from this scale as “a series of face-valid independent measures” and the summed score for these items can be interpreted as a composite score reflecting the level of direct and indirect exposure.

#### Family Resilience Assessment Scale

Family resilience was measured using the FRAS, a 54-item questionnaire designed and validated to measure distinct family processes (Sixbey, 2005; Walsh, 2006), based on Walsh’s (2006) Family Resilience Framework. The instrument has been validated in Singapore with good internal consistency and construct validity (Chew and Haase, 2016), yielding a 7-factor model with the following factors: (1) meaning-making and positive outlook, (2) transcendence and spirituality, (3) flexibility and connectedness, (4) resources-community, (5) resources-neighbors, (6) clarity and open emotional expression, and (7) collaborative problem solving. The items are rated on a 4-point Likert scale (1 = “Strongly disagree” to 4 = “Strongly agree”). Higher scores on the questionnaire indicate higher levels of family resilience.

### Data Analysis

Descriptive statistics and ICC were performed using IBM SPSS 26.0 statistical software. Descriptive statistics were reported as means with standard deviations. To test the first hypothesis, ICC were used to examine the agreement of scores within family dyads. ICC estimates and their 95% confidence intervals were calculated, based on a mean-rating (*k* = 2), absolute-agreement, and 2-way random effects model. Values were interpreted as follows (Koo and Li, 2016): ICC < 0.50, poor; 0.50 ≤ ICC < 0.75, moderate; 0.75 ≤ ICC < 0.90, good; and ICC > 0.90, excellent agreement.

To test the second and third hypotheses, we used the actor-partner interdependence model (APIM) framework with multilevel modeling, because it is a statistical technique that accounts for dependencies within dyadic data (Kenny et al., 2006). It treats measurements for individuals as nested within dyads and allows us to assess the contribution of either member of the dyad to the outcome variables (actor and partner effects). A person’s outcome can be due to his/her own characteristics (actor effect) and/or due to the characteristics of the dyad partner (partner effect). Correlations between the independent variables are assessed to control for and estimate the actor and partner relationships, while correlations among the outcome residuals allow the control of additional sources of non-independence, such as family influences (Cook and Kenny, 2005). The APIM framework also has the flexibility of incorporating variables that vary within and/or between dyads (mixed variables are those that vary both within and between dyads). APIMs were performed using a web-based R program built with RStudio’s Shiny package, “APIM_MM” (Kenny, 2015).

Model 1 was specified as such: COVID-19 Perceived Threat was entered as an outcome variable, while COVID-19 Exposure and COVID-19 Financial Impact were entered as the predictors (mixed variables) with potential actor and partner effects. The mean score of Family Resilience for each dyad was taken as a between-dyad independent variable, supported by the high ICC found (Table 2). Specifying it as a mixed variable would result in high collinearity, which would compromise the analysis (Kenny, 2015). Other covariates were the sociodemographic variables of age (mixed variable), gender (mixed variable), and housing type (between-dyad variable). These demographic variables were selected as covariates, because they have been found to factor in psychological responses to COVID-19 (Petzold et al., 2020; Bueno-Notivol et al., 2021; Nagasu et al., 2021). Housing type was used as a proxy for family-level socioeconomic status, because it is correlated with household income, it is often used as a surrogate of income status in Singapore (Ng et al., 2014), and it is also a social determinant of health (Low et al., 2016). Other indicators of socioeconomic status (household income and education level) were unsuitable in this study: firstly, the data for household income had discrepancies within dyads, as participants completed the survey independent of the other member and responses were based on each member’s assumption of the household income. It is possible that there was under- or over-reporting of household income as family members may not have fully shared their salaries with one another or members may have been unaware of income loss within the family caused by the pandemic. Nonetheless there was significant positive association (*χ*^2^ = 92.229, df = 9, *p* < 0.001) between household income (as reported by the participants) and housing type, further supporting housing type as an indicator of socioeconomic status. Second, the highest education level was not appropriate given that some participants had not yet completed their education and it would also not be a family-level indicator of socioeconomic status.

**Table 2:** Intraclass correlations (ICC) for dyad scores.

Measure	ICC(2,2) values	95% (CI)	*F*-test			
			Value	df1	df2	value of *p*
COVID-19 threat perception	0.58	(0.45–0.68)	2.40	199	199	< 0.001
COVID-19 psychological impact	0.44	(0.27–0.58)	1.82	199	199	< 0.001
COVID-19 financial impact	0.48	(0.31–0.60)	1.95	199	199	< 0.001
COVID-19 exposure	0.36	(0.16–0.52)	1.58	199	199	= 0.001
Family resilience	0.74	(0.66–0.81)	3.98	199	199	< 0.001

*Numbers rounded to 2 decimal places*.

Model 2 was similar to Model 1 in all respects, except for the outcome variable of the psychological impact of COVID-19. The equations of the models can be found in the Supplementary Material. The multilevel modeling was based on generalized least squares analysis with correlated errors and restricted maximum likelihood estimation.

The dyads were specified as indistinguishable since we were interested *a priori* in family group membership and sought to test the interdependence of COVID-19 psychological responses from the same household, and not from the influence of specific roles of family member. We therefore recruited a broad spectrum of family dyads and there were assorted relationships between the dyad members that cut across age, gender, and role (Table 3). We nonetheless ran tests of distinguishability to confirm our choice of specifying indistinguishable dyads. For Model 1, the test of overall distinguishability was not statistically significant (*χ*^2^ = 7.581, df = 6, *p* = 0.270) and the same was observed for Model 2 (*χ*^2^ = 3.393, df = 6, *p* = 0.758). As there was no statistical evidence for distinguishability in either model, we proceeded with the analyses based on indistinguishable dyads. Except for dummy variables, data were grand centered around each variable’s mean.

**Table 3:** Sociodemographic characteristics of study participants vs. population statistics.

	Study		Population
	*n* dyads	%	%
Dyad-Level			
*Relationship of family dyads*			
Parent–Child	86	43.0	
Couple	75	37.5	
Siblings	38	19.0	
Aunt-Nephew	1	0.5	
*Housing type*			
Public Housing (small): 1- to 2-room flats	2	1.0	6.5
Public Housing (medium): 3- to 4-room flats	102	51.0	49.3
Public Housing (large): 5-room flats and Executive Flats	62	31.0	22.9
Private Housing: apartments and landed properties	34	17.0	21.0
*Household income*			
$0 - $7,500^a^	101	50.5	56.1
Above $7,500^a^	99	49.5	43.9
Individual-Level	***n***	***%***	***%***
*Age*			
15–29 years	102	25.5	18.5
30–59 years	241	60.3	44.8
60 years old and above	57	14.3	22.2
*Gender*			
Female	251	62.8	51.1
Male	149	37.3	48.9
*Race*			
Chinese	346	86.5	74.4
Malay	24	6.0	13.5
Indian	25	6.3	8.9
Other	5	1.3	3.2
*Marital status*			
Single	150	37.5	31.2
Married^b^	228	57.0	59.9
Divorced/ Separated	10	2.5	3.9
Widowed	12	3.0	5.0
*Highest education level*			
Tertiary	269	67.3	32.4
Pre-Tertiary	62	15.5	24.9
Secondary	51	12.8	17.2
Primary	18	4.5	25.5
*Current occupation*			
Employed (includes self-employed)	274	68.5	62.2
Unemployed (includes students, home-makers, retirees)	126	31.5	37.8

*Numbers rounded to 1 decimal place. Population statistics were referenced from the following sources:**singstat.gov.sg**(2019/2020)*,*mom.gov.sg**(2020), and**data.gov.sg**(2018)*.

a *Population statistics threshold is $8,000*.

b *Includes 1 engaged couple living in the same household*.

## Results

### Sociodemographic Characteristics

Table 3 reports on the sociodemographic characteristics of the participants on two levels: the dyad and the individual. Comparisons were made with the population statistics where appropriate, in order to allow assessment of the representativeness of the study.

The majority of the dyad pairs had a parent–child relationship (43.0%), followed by couple (37.5%), sibling (19.0%), or other (0.5%) relationships. Comparable to the population distribution, approximately half of the dyads lived in medium-sized public housing (51.0%). In the reporting of household income, we noted that 18% of dyads had discrepancies, which is not entirely surprising, as not every member would be privy to income information. As such, we used broad household income categories of $0–7,500 and > $7,500, and the results largely followed the population trend (median household income in 2020 was $7,744; Singstat, 2020) with approximately half in each category.

The participants’ mean age was 42 years (SD = 15.18; range 15–85 years old), 62.8% were female, 86.5% were Chinese, and 67.3% had tertiary education. Overall, there was overrepresentation of the middle-aged, females, and those of higher education levels. The latter was much higher compared to the general population, where only approximately a third have tertiary education. This could have been partly due to the online recruitment, which may have excluded those unfamiliar with the Internet, such as those with less education and access to technology. There were no missing data in the dataset, as the participants would be prompted by the survey system to fill in any missing responses before submitting the online survey.

### Intraclass Correlations for COVID-19 Psychological Responses, Exposure, and Family Resilience

Table 2 reports the ICC, rounded to 2 decimal places, for dyadic scores on COVID-19 threat perception, psychological impact, exposure, financial impact, and family resilience. Dyads had a moderate level of agreement for COVID-19 threat perception, given ICC (2,2) = 0.58 (95% CI: 0.45 to 0.68). There was however weak agreement for COVID-19 psychological impact: ICC(2,2) = 0.44 (95% CI: 0.27 to 0.58), COVID-19 financial impact: ICC(2,2) = 0.48 (95% CI: 0.31 to 0.60), and COVID-19 exposure ICC(2,2) = 0.37 (95% CI, 0.17 to 0.52). Therefore, Hypothesis 1 was supported for the variable of COVID-19 threat perception, but not COVID-19 psychological impact. For family resilience, the ICC(2,2) was 0.74 (95% CI, 0.66 to 0.81), indicating substantial convergence in the dyads’ ratings of family resilience.

### Actor-Partner Interdependence Model Analyses

Table 4 shows the standardized coefficients (*β*), 95% CI and *p* values of the APIM models. For Model 1, R^2^ for the full model was 0.276 (*χ*^2^ = 124.332, df = 10, *p* < 0.001). COVID-19 exposure level was a significant factor in COVID-19 threat perception (*β* = 0.222, *p* < 0.001), but only as an actor effect. There was no significant partner effect. Similarly, COVID-19 financial impact was a significant factor as an actor effect (*β* = 0.353, *p* < 0.001), but with no significant partner effect (*p* = 0.056). Family resilience had an insignificant negative association with COVID-19 threat perception (*β* = −0.097, *p* = 0.057). Compared to females, males rated lower COVID-19 threat perceptions (*β* = −0.115, *p* = 0.003). The other covariates (age and housing type) were not significant.

**Table 4:** Summary of APIM analyses for Model 1 and Model 2.

		Model 1: COVID-19 threat perception				Model 2: COVID-19 psychological impact			
		Variable	Effect
95% CI				95% CI			
		Lower	Upper	value of *p*	*β*	Lower	Upper	value of *p*	*β*
COVID-19 exposure	Actor	0.165	0.381	<0.001	0.222	0.061	0.193	<0.001	0.162
	Partner	−0.078	0.138	0.585	0.024	−0.090	0.041	0.463	−0.031
COVID-19 financial impact	Actor	0.371	0.620	<0.001	0.353	0.337	0.489	<0.001	0.463
	Partner	−0.003	0.246	0.056	0.087	0.035	0.188	0.004	0.125
Family resilience		−0.059	0.001	0.057	−0.097	−0.041	−0.009	0.003	−0.130
Age		−0.017	0.039	0.433	0.032	−0.037	−0.002	0.027	−0.089
Male		−2.075	−0.424	0.003	−0.115	−0.514	0.556	0.939	0.003
Public Housing (small)		−9.174	1.166	0.130	−0.076	−4.919	0.692	0.141	−0.063
Public Housing (big)		−0.543	1.795	0.294	0.055	−0.783	0.486	0.648	−0.021
Private Housing		−1.304	1.609	0.838	0.011	−0.727	0.853	0.876	0.007

*Public Housing (small): 1-room and 2-room flats; Public Housing (big): 5-room and “Executive Flats”; and Private Housing: apartments and landed properties. (Reference group: 3-room and 4-room public housing flats)*.

For Model 2, R^2^ for the full model was 0.363 (*χ*^2^ = 176.561, df = 10, *p* < 0.001). COVID-19 exposure level had a significant actor effect in COVID-19 psychological impact (*β* = 0.162, *p* < 0.001), with no significant partner effect. COVID-19 financial impact had significant actor (*β* = 0.463, *p* < 0.001) and partner effects (*β* = 0.125, *p* = 0.004). Family resilience was a significant factor, with higher family resilience associated with lower psychological impact (*β* = −0.13, *p* = 0.003). Participants who were older rated lower psychological impact (*β* = −0.089, *p* = 0.027). Gender and housing type were not significant covariates.

Given that there was a significant partner effect for COVID-19 financial impact on psychological impact (Model 2), the results partially supported Hypothesis 2. Hypothesis 3 was also partially supported—family resilience was negatively related to COVID-19 psychological impact, but not threat perception. Table 5 provides a summary of all the hypotheses and their results.

**Table 5:** Overview of hypotheses and results.

Hypotheses	COVID-19 psychological responses		COVID-19 threat perception	COVID-19 psychological impact
(1) Family dyad members will demonstrate at least moderate agreement (ICC(2,2) ≥ 0.5) on COVID-19 psychological responses.	Supported^***^	Not supported
(2) There will be partner effects of COVID-19 risk factors (exposure and financial impact) on a dyad member’s psychological responses.	COVID-19 Exposure: Not supported COVID-19 Financial Impact: Not supported (*p* = 0.056)	COVID-19 Exposure: Not supported COVID-19 Financial Impact: Supported^**^
(3) COVID-19 psychological responses will be negatively associated with the level of family resilience, after accounting for COVID-19 risk factors.	Not supported (*p* = 0.057)	Supported^**^

*** *p < 0.001 and*

** *p < 0.01*.

### *Post-hoc* Analyses

Given that the construct of family resilience comprises seven factors as validated in the local population (Chew and Haase, 2016), we assessed the contribution of each of the factors in the models by substituting the overall measurement for family resilience with each factor’s measurement. (As the factors were correlated with each other, analyzing them as a set of predictors would result in multicollinearity.) Due to the multiple comparisons, we reduced the α threshold from 0.05 to 0.007 using the Bonferroni correction. At this threshold, the factors of “meaning making and positive outlook” (*β* = −0.159, CI: −0.164 to −0.051, *p* < 0.001) and “flexibility and connectedness” (*β* = −0.126, CI: −0.201 to −0.038, *p* = 0.004) were significant in Model 2. In the same model, the factors of “clarity and open emotional expression” and “collaborative problem solving” had value of *p*’s ranging between 0.007 and 0.05, while the other factors had values of *p* > 0.05. All the family resilience factors in Model 1 had values of *p* > 0.05.

Although the ICC analyses pointed toward the utility of averaging the family resilience scores between dyad members to use as a between-dyad variable, we performed further analyses to test if the Model 1 and Model 2 results would hold if individual ratings for family resilience were to be used as predictors instead. The same pattern of results was observed: In Model 1, there were significant actor effects for COVID-19 exposure and financial impact. In Model 2, there was a significant actor effect for COVID-19 exposure, while COVID-19 financial impact had both actor and partner effects. Family resilience had a significant negative actor effect, but no significant partner effect (although this result is compromised by the collinearity). The covariate results remained the same in both models. Therefore, the findings of the analyses with family resilience as a between-dyad variable can be used with confidence.

## Discussion

In this dyadic cross-sectional study, we assessed three aspects relevant to the family’s psychological responses (threat perception and psychological impact) to the COVID-19 pandemic. (Table 5 summarizes the outcomes.) Concerning the first research question, we found concordant dyad responses for the threat perception of COVID-19, but not for psychological impact. On the second research question, the significant partner effect found was that dyad members’ financial difficulties mutually affected their experience of psychological impact. The other partner effects were not significant. Regarding the third research question, we found that family resilience was had a significant association with lower psychological impact of COVID-19, but not in threat perceptions. Complementing the mental health studies on individuals during the pandemic, this study provides novel data on family-level psychological responses and the influence of personal risk factors on other family members. Importantly, our data also point to the protective role of family resilience in mitigating the psychological impact of the pandemic, suggesting avenues for future crisis preparation.

### COVID-19 Psychological Responses as Dyadic Responses

As would be predicted by family theory (Hess and Handel, 1959; Reiss and Oliveri, 1983; Kerr and Bowen, 1988), we hypothesized and found in this study that families demonstrated statistically significant agreement in their perception of threats, specifically fear and stress due to COVID-19. This result echoes the correlated fear of the coronavirus observed between husbands and wives (Ahorsu et al., 2020). The perception of threats has been attributed to “bottom-up” processing of stimuli in the environment (e.g., crowded conditions) that is guided by “top-down” processing, driven by contexts, and expectations (e.g., knowledge of prevalence of infections and expectation of encountering an infected case; Sussman et al., 2016). Families living together are likely to encounter similar environmental stimuli and discuss their expectations, thereby developing similar threat perceptions. Shared family perceptions can be seen as family views and interpretations that have been “collectively constructed by family members as they interact with each other” (Patterson, 2002). These collective views may also be more than simple agreement between family members, who can share explanatory systems that generate a shared reality (Reiss and Oliveri, 1983). In a study of emotional affect among family members, small correlations were found (<0.4) when they were together, and there were no significant correlations in global emotional patterns when the family was not together (Larson and Richards, 1994). This suggests a spatio-temporal element and the importance of the shared environment, such as living in the same household, and especially during the pandemic when people spend more time with each other at home. In our study, although threat perception had concordance within dyads living in the same household, it was weaker for the outcome of COVID-19 psychological impact (ICC = 0.44). This finding for psychological impact reflects the modest correlations in emotional affect among family members found in Larson and Richards’ (1994) study, suggesting that these psychological attributes may be shaped by factors beyond group settings. As observed by studies during the pandemic, part of the variability for psychological responses to COVID-19 may be explained by differences in individual-level factors, such as lifestyle-related factors (e.g., sleep; Nagasu et al., 2021), individual resilience (Coulombe et al., 2020), financial stressors (Xiong et al., 2020), and occupational risks related to virus exposure (Brooks et al., 2018).

### Risk Factors and Their Effects Within the Family Dyad

The financial impact of COVID-19 demonstrated strong actor and partner effects on the outcome of COVID-19 psychological impact, meaning that financial impacts felt by one dyad member influenced not just his/her own psychological state (actor effect), but also the other dyad member’s psychological state. [There was also a strong actor effect in the outcome of COVID-19 threat perception, but an insignificant partner effect (*p* = 0.056)].The significant actor effects on psychological impact support the findings of studies in individuals, where unemployment and loss of family income have been associated with poorer mental health (Qi et al., 2020; Xiong et al., 2020). As for the significant partner effects, they corroborate statistically what has been observed through a qualitative survey about how one family member’s job loss or income instability during the pandemic can create stress among other family members, even for middle to high income families (Carroll et al., 2020).

Contrary to our hypothesis, the risk factor of COVID-19 exposure levels demonstrated no significant partner effect for both outcomes of COVID-19 threat perception and psychological impact. This result differs from other studies: Ying et al. (2020) observed that the occupational risk of an individual does not just impact personal mental health, but also appears to affect the mental health of the person’s family, as people who have family members in high-risk occupations (e.g., healthcare frontline work) have reported more anxious and depressive symptoms. Similarly, Tanoue et al. (2020) found that if an individual had a family member who tested positive for COVID-19, higher levels of psychological distress were reported in that individual. One potential explanation could be the timing of the study, which occurred during a relatively stable period with low community transmission, when people’s worry about the contagion could have abated, as contrasted with the studies that were performed during the initial escalating phases of the pandemic (Tanoue et al., 2020; Ying et al., 2020). In line with the explanation, our participants rated low on items that concerned COVID-19 diagnosis, coronavirus-like symptoms, or being in proximity to people diagnosed or exhibiting symptoms (the mean score among these items was 1.25 on a 7-point Likert scale, where 1 represents “not true of me at all”). Furthermore, we used the APIM framework, which teases out the different sources of correlations using the predictor and outcome variables for both dyad members, whereas the abovementioned studies (Tanoue et al., 2020; Ying et al., 2020) associated the predictor from one member to the outcome from the other member.

Although COVID-19 exposure did not have significant partner effects on the dyad member’s psychological responses, it had a significant actor effect on one’s own psychological responses. Beyond direct experiences with COVID-19 relating to personal infection or that of contacts, frequent exposure to the news concerning COVID-19 appears to be a risk factor for psychological distress (Lee et al., 2020; Xiong et al., 2020) and conversely, spending less time on health information was associated with less mental health impact (Wang et al., 2021). Overall, one’s mental health state is consistently affected by personal risk exposure (Benke et al., 2020; Ettman et al., 2020; Lai et al., 2020; Rossi et al., 2020; Wang et al., 2020; Zhang et al., 2020) and should be accounted for in analyses of mental health impact (Ding et al., 2021), as also shown by the significant actor effect of COVID-19 exposure in our study.

### Family Resilience as a Factor in the Psychological Impact of COVID-19

After accounting for sociodemographic effects and the risk factors of COVID-19 exposure and financial impact, we found that family resilience was significantly associated with lower COVID-19 psychological impact. A negative relationship was also seen with the outcome of COVID-19 threat perception, but the model was not significant (*p* = 0.057). Taken together, these findings partially supported our third hypothesis and contributes some empirical support for theories on employing family resilience frameworks in helping populations cope with crises like COVID-19 (Walsh, 2003; Masten and Motti-Stefanidi, 2020; Prime et al., 2020). Among the domains within the family resilience construct (Chew and Haase, 2016) that were significant, we found that “meaning making and positive outlook” and “flexibility and connectedness” were strong factors for reducing COVID-19 psychological impact. These findings point to specific aspects of family resilience that could be helpful in preparing the population for another crisis on the scale of COVID-19. According to the Walsh family resilience framework (Walsh, 2003), shared beliefs are at the core of a family’s resilience. In a situation like COVID-19, the making of meaning involves shared struggles to make sense of losses, put them in perspective and learn how to recover from those losses (Nadeau, 2008), in order to restore order, meaning, and purpose in life (Walsh, 2020). This is layered with a positive outlook or hope, which is not simply “relentless optimism or good cheer” (Walsh, 2020), rather it can be seen a process of mutual encouragement among family members to persevere and take initiative, and at the same time reinforcing shared pride and a “can do” spirit (Walsh, 2020). The processes of flexibility and connectedness foster resilience in the organizational patterns of family functioning (Walsh, 2003), because in the post crisis period, it may be difficult for families to return to the previous way of life and they must find ways to cope with the “new normal.” An example of flexibility in family organizational patterns would be ability to alter family leadership (Walsh, 2003), e.g., if the head of the household were to fall ill, flexibility in roles would allow the wife or children to step up to lead instead. During this time, connectedness or cohesion is also important, such as mutual support, collaboration, and commitment to go through the changes and difficulties together (Walsh, 2003).

While statistically significant, the effects of family resilience were small (ranging from −0.1 to −0.16), and total variance explained by the models was 28 and 36%, respectively, which points to the likely contribution of other factors. Other risk factors specific to mental health during COVID-19 include the presence of chronic illness (Xiong et al., 2020), while protective factors identified include confidence in doctors, self-efficacy, hope, trait resilience, and family support (Coulombe et al., 2020; Petzold et al., 2020; Ding et al., 2021; Lim et al., 2021a,b; Wang et al., 2021).

### Limitations

Due to the COVID-19 situation, recruitment and data collection were conducted online. This limited participation to those with Internet access and may also have led to an overrepresentation of sociodemographic groups, such as those with tertiary education, middle-aged, and female, thus affecting the generalizability of the findings to the local population.

We assumed that family dyads were adequate representations of the family. Sampling from the entire family would be ideal, though this would be difficult to achieve due to eligibility, consent, and data requirements from every member. The current APIM framework is well suited to dyadic data. Having different sizes of family groups would require more complex multilevel modeling.

As with cross-sectional studies, causal attributions are cautioned. Nonetheless a capacity like family resilience requires time to develop (Walsh, 2003) and therefore suggests that the measured variable of family resilience had temporal precedence to the outcome variables (psychological responses to COVID-19) in this study.

### Concluding Perspectives

Taken together, the findings show that although individual factors have significant influence (e.g., as seen through actor effects), there may also be benefit in developing interventions that address risk and protective factors in the family, given the partner effects in the risk factor of financial impact, and the protective factor of family resilience in the psychological impact of COVID-19. Family interventions can be relevant beyond mental health. For example, while public health measures like social distancing, handwashing, and wearing masks may be mandated by the authorities, ultimately “they are implemented at the family and individual level, with cascading consequences for hospitals and population survival” (Masten and Motti-Stefanidi, 2020). There may thus be value in addressing the family for behavioral interventions. Overall, there is a need for an ecological systems perspective to address multilevel risk and protective factors, which may help prepare the population for future crises or pandemics (Coulombe et al., 2020).

## Data Availability Statement

The raw dataset for this study will not be readily available, because of the stipulations from the local institutional review committee on data sharing beyond the approved study team members. Requests to access the datasets should be directed to Y-CH, gmshycl@nus.edu.sg.

## Ethics Statement

The studies involving human participants were reviewed and approved by SingHealth Centralised Institutional Review Board (CIRB), SingHealth Duke-NUS Academic Medical Centre. Written informed consent from the participants’ legal guardian/next of kin was not required to participate in this study in accordance with the national legislation and the institutional requirements.

## Author Contributions

Y-CH conceptualized and designed the study. JT supervised the study. Y-CH and JT acquired funding for the study. MC performed the project administration. MC and Y-CH performed the data analysis and wrote the first draft, while MC and DM did the data charting and visualization. All authors contributed to manuscript revision, read, and approved the submitted version.

## Funding

This study was funded by a grant (CGDec19S11) from the SingHealth Regional Health System (PULSES) Centre Grant, Singapore. Y-CH, MC, and DM were funded by a grant (NMRC/CG/C027/2017) from the National Medical Research Council, Singapore. The funders had no other role in this study.

## Conflict of Interest

The authors declare that the research was conducted in the absence of any commercial or financial relationships that could be construed as a potential conflict of interest.

## Publisher’s Note

All claims expressed in this article are solely those of the authors and do not necessarily represent those of their affiliated organizations, or those of the publisher, the editors and the reviewers. Any product that may be evaluated in this article, or claim that may be made by its manufacturer, is not guaranteed or endorsed by the publisher.
