# Supplementary material for: Family Resilience and Psychological Responses to COVID-19: A Study of Concordance and Dyadic Effects in Singapore Households
Source: Front Psychol. 2022 Mar 1;13:770927. doi: 10.3389/fpsyg.2022.770927 (PMC8923423; doi:10.3389/fpsyg.2022.770927)
Supplement: Supplementary file 1 [file Data_Sheet_1.docx]

Supplementary Material

# Equations for the APIM models using multilevel modelling

We present below the equations for Model 1, which models the outcome of COVID-19 Threat Perception using the independent variables of COVID-19 Exposure, COVID-19 Financial Impact, Family Resilience and the covariates of Age, Male, Public Housing (small), Public Housing (big) and Private Housing.

As the APIM models (Models 1 and 2) were estimated using multilevel modelling, there were two ‘levels’ of equations:

The **Level-1 equation** (individual-level) is a fixed-effects, linear model that predicts the COVID-19 Threat Perception for each individual member *j* in dyad *i* as a function of the dyad-specific intercept and slopes plus an error term.

$${ThreatPerception}_{ij}= \beta_{0,i}+\beta_{1,i}\left( Exposure_{actor} \right)_{ij}+\beta_{2,i}\left( Exposure_{partner} \right)_{ij}+\beta_{3,i}\left( FinancialImpact_{actor} \right)_{ij}+\beta_{4,i}\left( FinancialImpact_{partner} \right)_{ij}+\beta_{5,i}\left( Age \right)_{ij}+\beta_{6,i}\left( Male \right)_{ij}+e_{ij}$$

The **Level-2 equations** (dyad-level) express the dyad-specific intercept and slopes as functions of the **between-dyad variables** (Family Resilience, and the dummy variables for housing). The equation for the intercept, $\beta_{0,i}$ includes a random effects component, $f_{i}$, which estimates the degree to which COVID-19 Threat Perception varies from dyad to dyad after controlling for the level-1 predictors and covariates. The equations for the slopes ($\beta_{1,i} to \beta_{6,i}$) do not include random effects, because with only two data points from each dyad, there is a limitation on allowing the slopes to vary from dyad to dyad. The slopes thus have to be constrained to be equal across dyads, but this does not affect the estimates of the multilevel model, as tests of the null hypothesis are not biased by having different slopes (Kenny et al., 2006).

$$\beta_{0,i}=\alpha_{0}+\alpha_{1}\left( FamilyResilience \right)_{i}+\alpha_{2}\left( PublicHousing\_small \right)_{i}+\alpha_{3}\left( PublicHousing\_big \right)_{i}+\alpha_{4}\left( PrivateHousing \right)_{i}+f_{i}$$

$$\beta_{1,i}=\gamma_{0}+\gamma_{1}\left( FamilyResilience \right)_{i}+\gamma_{2}\left( PublicHousing\_small \right)_{i}+\gamma_{3}\left( PublicHousing\_big \right)_{i}+\delta_{4}\left( PrivateHousing \right)_{i}$$

… and so on until $\beta_{6,i}$

The Model 2 equations are exactly the same as the Model 1 equations, save for a simple substitution of the outcome variable of COVID-19 Psychological Impact.
